# Rapid tropicalization evidence of subtidal seaweed assemblages along a coastal transitional zone

**DOI:** 10.1038/s41598-023-38514-x

**Published:** 2023-07-20

**Authors:** Jonas de Azevedo, João N. Franco, Cândida G. Vale, Marco F. L. Lemos, Francisco Arenas

**Affiliations:** 1 CIIMAR - Centro Interdisciplinar de Investigação Marinha e Ambiental, Terminal de Cruzeiros do Porto de Leixões, University of Porto, Matosinhos, Portugal; 2 Departamento de Biologia, Faculdade de Ciências, Universidade do Porto, Porto, Portugal; 3 MARE – Marine and Environmental Sciences Centre & ARNET – Aquatic Research Network, ESTM, Polytechnic of Leiria, 2520-641 Peniche, Portugal

**Keywords:** Ecology, Environmental sciences, Ocean sciences

## Abstract

Anthropogenic climate change, particularly seawater warming, is expected to drive quick shifts in marine species distribution transforming coastal communities. These shifts in distribution will be particularly noticeable in biogeographical transition zones. The continental Portuguese coast stretches from north to south along 900 km. Despite this short spatial scale, the strong physical gradient intensified by the Iberian upwelling creates a transition zone where seaweed species from boreal and Lusitanian-Mediterranean origin coexist. On the northern coast, kelp marine forests thrive in the cold, nutrient-rich oceanic waters. In the south, communities resemble Mediterranean-type seaweed assemblages and are dominated by turfs. Recent evidence suggests that in these coastal areas, marine intertidal species are shifting their distribution edges as a result of rising seawater temperatures. Taking advantage of previous abundance data collected in 2012 from subtidal seaweed communities, a new sampling program was carried out in the same regions in 2018 to assess recent changes. The results confirmed the latitudinal gradient in macroalgal assemblages. More importantly we found significant structural and functional changes in a short period of six years, with regional increases of abundance of warm-affinity species, small seaweeds like turfs. Species richness, diversity, and biomass increase, all accompanied by an increase of community temperature index (CTI). Our findings suggest that subtidal seaweed communities in this transitional area have undergone major changes within a few years. Evidence of “fast tropicalization” of the subtidal communities of the Portuguese coast are strong indication of the effects of anthropic climate change over coastal assemblages.

Change is a characteristic of our Earth through its history but current anthropogenically driven climate change has pushed the oceans well outside their historical limits^1^. Current ocean temperature exceeds the values recorded over the past 400,000 years, increasing at a rate of 0.88 °C per decade, in the last 40 years^2^. The sea water temperature is recognized as the main driver of key physiological processes in marine ectotherms. Species are often adapted to optimum temperature ranges, occupying restricted thermal niches along latitudinal temperature gradients^3,4^. In marine ecosystems, where high velocities of climate-change exceed those in the terrestrial ecosystems, marine isotherms move very fast (ca. 21.7 km/decade^5^). Thus, to avoid local extinction, marine species are forced either to adapt to new conditions or to track their climatic niches in space or time, i.e., spatial and phenological shifts^6^, triggering fast compositional changes^7^. Globally, the observed trends are poleward range shifts, resulting in an increase of species at higher latitudes^7^. In temperate ecosystems, the outcome is a rising proportion of species with tropical affinity, a process named ‘tropicalization’ in marine habitats^6,8^. Tropicalization phenomenon is profusely reported in marine literature^9,10^. This phenomenon is often described by using the community temperature index (CTI), which is the average of species’ thermal affinities weighted by their abundance in the community^11,12^. CTI changes reflects differences in the community composition and species dominance. This measure allows tracking shifts in communities due to thermal changes by the relative contribution of each species with a given thermal affinity^12^.

The Portuguese continental coast is a biogeographic transition zone, harbouring fauna and flora with cold and warm-water temperature affinities^13–16^. The Iberian upwelling system (active from 37º N to 43º N) affects the coastal area, having large effects on its oceanographic conditions^17,18^. The upwelling system creates unique conditions for the persistence of species with cold-water affinity^19,20^. The intensity of the upwelling follows a latitudinal pattern, being stronger on the North coast of Portugal and Spain which results in a highly productive ecosystem with low sea surface temperature and nutrient-rich seawater nearshore, even in summer^21,22^. In the southern continental coastal area of Portugal, seawater is warm, poorer in nutrients, and has a strong Mediterranean influence^23^. Contrary to other eastern boundary upwelling systems, the intensity of the Iberian upwelling is predicted to decrease in the future^17,22,24^. This upwelling weakening may result in an increase in the sea surface temperatures along the Portuguese continental coast^25,26^ and a reduction in the surface input of nutrients in the nearshore^27^.

Regardless of the intensity and direction of the ocean climate changes in the Atlantic seashores of the Iberian Peninsula, there is growing evidence that coastal communities are undergoing rapid transformations. While these community changes may involve the replacement of certain species by others that perform similar roles and functions, allowing for functional redundancy^28^, we contend that this is not the situation along the Portuguese continental coast. For instance, there is a documented increase in the abundance of marine species with an affinity for warm seawater^25,29–31^, along the Iberian coast. Conversely, certain habitat forming seaweed species, such as kelps from the genus Laminariales and Tilopteridales, are experiencing a decline^29,32–35^. Kelps are vital components of subtidal and intertidal communities in temperate reefs. Functioning as “engineering species”, they create marine forests with a complex three-dimensional structure that significantly influences the physical, chemical, and biological dynamics of the ecosystem. These forests provide shelter and nursery habitats for multiple species^36^. Moreover, kelp forests support artisanal fisheries^37^ and contribute to carbon sequestration^38,39^.

In fact, most kelp species thrive in cold, nutrient-rich seawaters^40,41^, and are highly vulnerable to warming oceans, particularly in regions near their southern limits, such as in Portugal^42^. The decline in habitat-forming species often creates the opportunity for proliferation of more opportunistic assemblages, like turf species, which tend to increase in abundance and dominate seaweed communities^43^. However, turf species create a smaller and less complex three-dimensional structure, resulting in a more homogeneous ecosystem with reduced available resources and functional diversity^44–48^.

The general objective of this work is to understand the magnitude and direction of recent changes on the subtidal seaweed communities along the Portuguese coast while examining evidence of tropicalization or other climate change-linked shifts. Although there is ample research on intertidal species in the Portuguese coastal area^14,15^, limited information is available on subtidal coastal assemblages, particularly regarding seaweed communities^16,49^. Leveraging a previous sampling survey, we conducted an assessment of changes occurring in the subtidal seaweeds assemblages over a period of six years (2012–2018) across different areas along the latitudinal gradient of the continental Portuguese coastline. We aimed to answer the following questions: Are there temporal differences in the structure of seaweed communities across this latitudinal gradient? If changes in the communities’ composition are observed, what is their nature? And finally, are changes linked with the species thermal affinities? Given the current scenario of fast seawater warming and the accelerated pace of changes^5^, we hypothesize that alterations in the structure of the algal assemblages are detectable even within this relatively time frame. Furthermore, we anticipate shifts in the communities’ thermal affinities, including an increase in the abundance of warm-water affinity species, and a potential reduction of cold- water affinity species. Finally, we expect that these changes will lead to measurable functional changes in the communities, including an enhanced abundance of turf species and a reduction in the abundance of canopy species.

## Methods

### Study area

**Figure 1:**
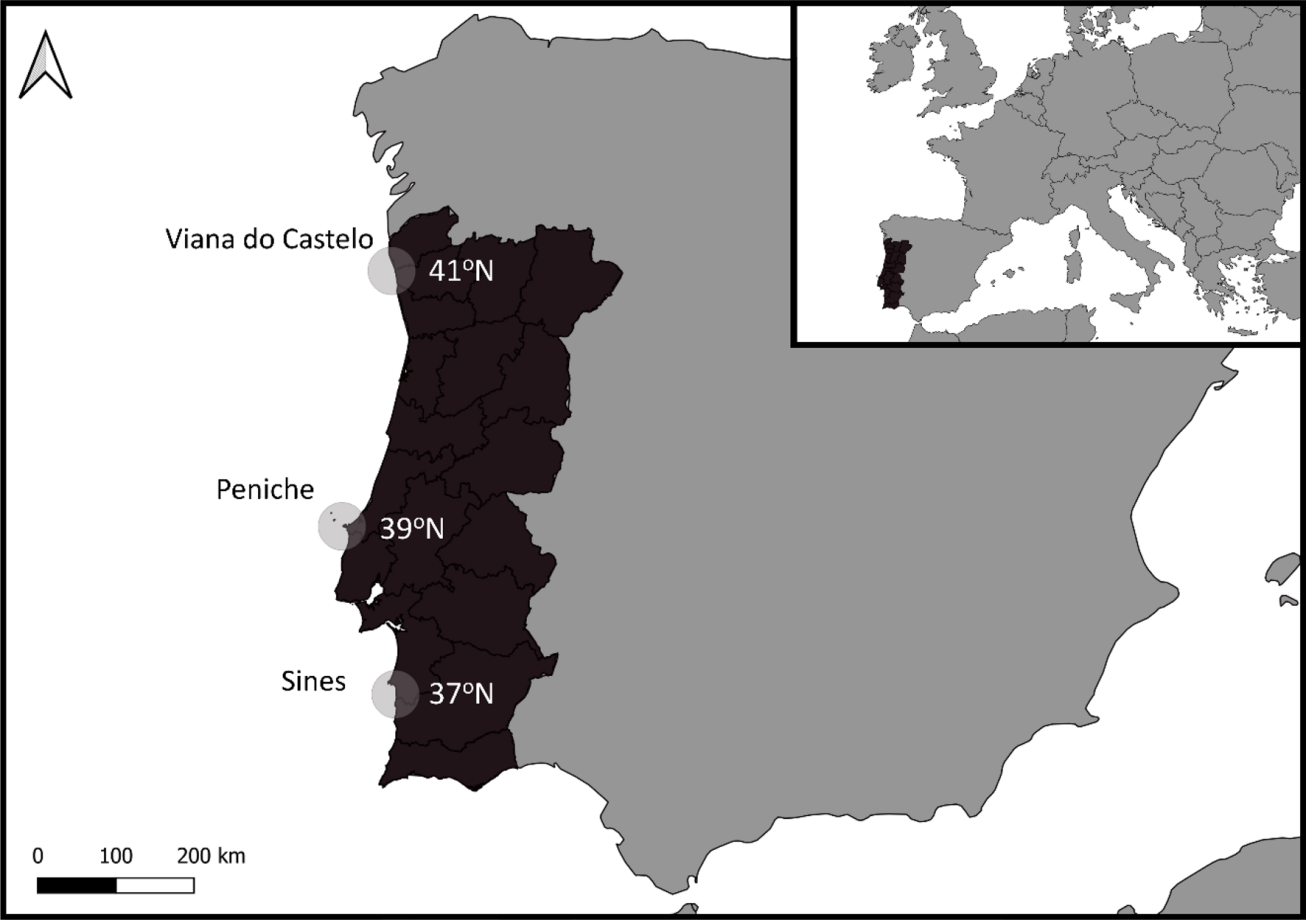
Map of Portugal continental showing the location of the three study regions: Viana do Castelo (41° N), Peniche (39° N) and Sines (37° N) (QGIS 3.28.3-Firenze, shapefile- https://gisco-services.ec.europa.eu/distribution/v2/countries/download/ref-countries-2020-01m.shp.zip).

Following the survey design of Tuya et al.^16^, three regions were surveyed along the continental coast of Portugal: Viana do Castelo—41°N, (VIA), Peniche—39.2°N (PEN), and Sines—37.8°N (SIN) (Fig. 1). The coast of Portugal continental has a north-to-south orientation and the selected regions have a similar wave exposure to the dominant NW and W swells. This coastline is characterized by extensive sandy beaches interspersed with limestone, sandstone, shale, or granitic reefs in both the intertidal and the shallow subtidal^16^. At each region, five different subtidal reefs were sampled using a protocol similar to Pinho et al.^49^ and detailed below.

### Collection and data analyses

Surveys were conducted during six diving campaigns carried out in the summers of 2012 and 2018. For each survey campaign and within each region (Viana do Castelo, Peniche and Sines), five rocky reef areas located one to five km apart and at depths between 6 and 12 m were randomly selected. Scientific diving techniques were utilized to sample the selected areas. Within each reef, six quadrats of 50 × 50 cm (0.25 m^2^) were haphazardly placed on the top of horizontal reefs bottom. All macrophytes present were carefully scraped and collected in textile bags. The samples were appropriately labelled and transported to the laboratory in cool boxes and frozen until processing. Seaweeds within each sample were identified to the lowest feasible taxonomic level, mostly species, and their weight was determined after excess water was removed through shaking. Species richness was determined as the total count of distinct species in the samples, while the Shannon H' diversity index, incorporated both species richness and evenness to assess the diversity and distribution of the species within the community^45^. Finally, to perform a morpho-functional analysis of the samples, the seaweed species were categorized into four morpho-functional groups: ‘encrusting’, ‘turf-forming’, ‘sub-canopy’, and ‘canopy’, following the algal morpho-functional classifications described by Arenas et al.^45^.

A detailed quantitative assessment of the thermal shifts was done using the community temperature index (CTI), which has been used to assess the thermal shift of communities and as an indicator of climate change^50,51^. The CTI is the weighted mean of species’ thermal preferences calculated for a community^11^. The temperature preference of species is given by the species temperature index (STI), which is the 50th percentile (median) of temperatures experienced throughout the species’ area of occupancy. To assess each species’ probable area of occupancy, and the respective profile of the temperature experienced, ecological niche models were implemented (detailed description in “Supplementary Material”). Only taxa identified at the species level were used in this analysis. Presence data were retrieved from OBIS (https://obis.org/) and GBIF (https://www.gbif.org/) public databases, and pruned to remove misidentifications, possible introductions as well as remove duplications, and data clustering (details in “Supplementary Material”). Uncorrelated environmental variables (Table S1) were retrieved from Bio-Oracle (https://www.bio-oracle.org/) at 5 arcmin of resolution (c. 9.2 km at the equator). Models were built using the Maximum Entropy approach^52^, with default parameters, 5 replicates, and 25% of the data for testing. To assess the global area of occupancy of each species, continuous models were converted into binary maps using a threshold that maximizes both numbers of presences and absences correctly classified by the model^53^. To define the thermal “niche” of each species, the maps of the area of occupancy were used to extract the quantiles of the sea surface temperature, for each species^11^. The median (50th percentile) of temperatures in each species occupied areas was used as the STI^11^. The Community Temperature Index (CTI) was calculated for each locality, rocky reefs, and time (all diving campaigns) as the abundance-weighted mean of all STIs^12^. Finally, the four processes promoting changes in CTI were assessed following the methodology of McLean et al.^54^, where (i) tropicalization is an increase in the abundance of warm-affinity species; (ii) borealization is an increase in abundance of cold-affinity species; (iii) de-tropicalization is a decrease in the abundance of warm-affinity species; (iv) and de-borealization is a decrease in the abundance of cold-affinity species). Here, warm-affinity species exhibit STIs higher than the mean of the community, while cold-affinity species have STIs lower than the mean^54^.

### Statistical analysis

Data were analysed using R software^55^ and plots were produced using the package ggplot2^56^. As the survey design followed Tuya et al.^16^, we replicated the analytical procedure but incorporated time as a factor to compare 2012 and 2018 data. Community level analyses were conducted using the vegan library^57^ and Primer v6^58^. Non-metric multi-dimensional scaling (NMDS) plots were used to visualize differences in algal assemblage composition among regions and times. Those differences were tested using a three-way permutational multivariate analysis of variance (PERMANOVA^59^) based on Bray–Curtis dissimilarities calculated from square-root transformed data. The PERMANOVA model included three factors: Time (two levels, fixed), Region (three levels, fixed and crossed with Time), and Reef (five levels, random and nested within both Time and Region). When tests were significant and for those relevant effects, post-hoc comparison tests were carried out to identify the nature of differences. To test for homogeneity of dispersion among our multivariate samples we ran a PERMADISP test using Primer.

Analyses of variance (ANOVA) on univariate community descriptors and CTIs were carried out using the GAD library^60^ and the same design used in Permanova. Before performing each ANOVA, the assumption of homogeneity of variances was checked using Cochran’s C test^61^. When the assumption was not achieved, data were transformed to avoid heterogeneous variances. ANOVAs on the abundance of functional groups were not homogeneous in all the analyses, however balanced ANOVAs are quite robust to departures of homoscedasticity^61^ and significant p values were smaller than 0.01, thus type I errors were improbable. When relevant, Student- Newman-Keuls (SNK) tests were used for a posteriori comparison of means.

## Results

In 2012 and 2018, a total of 105 taxa of macroalgae were identified from the 180 samples, including 95 species, 9 genera, and one taxon at family-level (complete list of observed macroalga available in Table S2). In the first survey (2012), 79 taxa were recorded, including 71 species and 8 genera. In 2018, we found a slightly larger number of taxa, with 84 taxa, including 77 species, 6 genera and one taxa at family-level. Among the reefs in the northernmost region (Viana do Castelo), *Laminaria hyperborea*, *Saccorhiza polyschides* and *Dilsea carnosa* were the most abundant species. In the central region of Peniche, the dominant species were the red seaweed *Plocamium cartilagineum* and two introduced seaweeds, *Asparagopsis armata* and *Symphyocladia marchantioides,* with the later only recorded in the 2018 survey. Finally, in the reefs of the South region, the most abundant species in the samples were *Asparagopsis armata*, *Sphaerococcus coronopifolius,* and *Codium adhaerens*.

**Figure 2:**
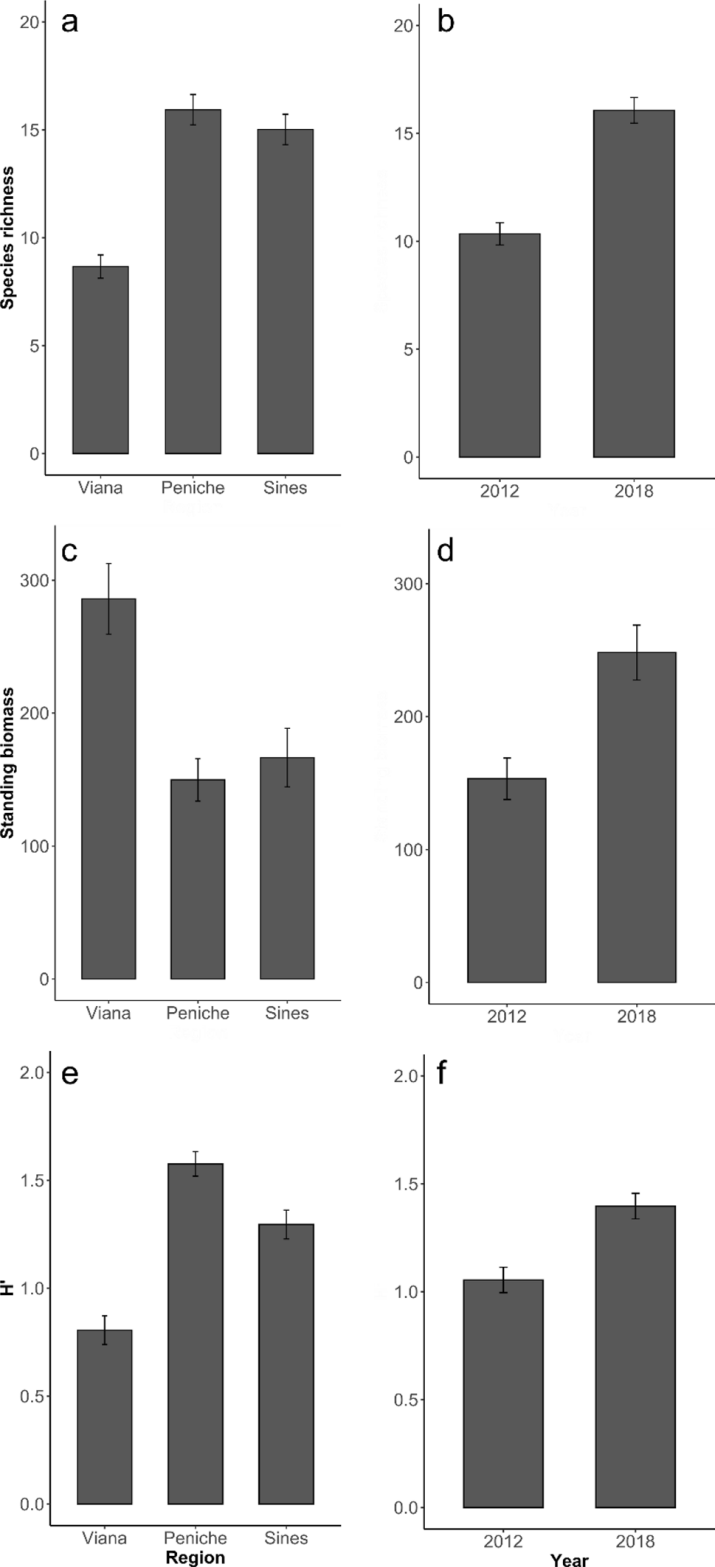
Bar plots to illustrate significant effects of region and time for (**a**, **b**) Species richness, (**c**, **d**) Algal standing biomass (wet weight g 0.25 m^2^) and (**e**, **f**) Shannon H’ diversity index. Bars are earned mean values ± SE, n = 6.

Spatial and temporal differences among assemblages were observed through the diversity of descriptors (Fig. 2). Seaweed species richness, assemblages’ biomass, and Shannon diversity indexes significantly increased between 2012 and 2018 (Table 1, Fig. 2a,d,f). No significant changes were observed in evenness between the two years. In terms of regional differences, Viana sites exhibited lower species richness and Shannon diversity indexes compared to Sines and Peniche (Table 1, Fig. 2a,e). Evenness values were higher in Peniche than in Viana, and median evenness in Sines (Table 1). Regional differences were also observed in standing crop biomass, with Viana reefs showing higher biomass than Sines and Peniche (Table 1, Fig. 2d). Finally, significant differences were found in all descriptors at the local scale (among reefs) (Table 1).

**Table 1:** ANOVA examining the effects of time, region and reef on the richness of algal taxa, total algal biomass, the Shannon H’ diversity index and the Pielou’s evenness index.

Source of variation	df	Taxa richness		Algal biomass		Shannon H´ index		Pielou’s evenness index	
		MS	*F*	MS	*F*	MS	*F*	MS	*F*
Time (T)	1	1584.2	25.970***	17.13	10.515**	6.93	10.266**	0.17	2.173
Region (R)	2	946.12	15.510***	12.12	7.438**	8.28	12.255***	0.52	6.402**
T x R	2	129.12	2.116	4.85	2.980	0.03	0.045	0.03	0.481
Reef (T x R)	24	61	6.168***	1.62	3.3141***	0.68	4.637***	0.08	4.278***
Residual	150	9.89		0.49		0.15		0.01	
Cochran C' test		C = 0.104, P = 0.19		C = 0.085, P = 0.66		C = 0.118, P = 0.073		C = 0.12117, P = 0.059	
Transformation		None		Log(x)		None		None	
SNK test		Time	Region	Time	Region	Time	Region	Region	
		2012 < 2018	VIA < PEN = SIN	2012 < 2018	PEN = SIN < VIA	2012 < 2018	VIA < PEN = SIN	VIA = SIN < PEN	

*p < 0.05, **p < 0.01, ***p < 0.001.Significant values are in bold.

**Figure 3:**
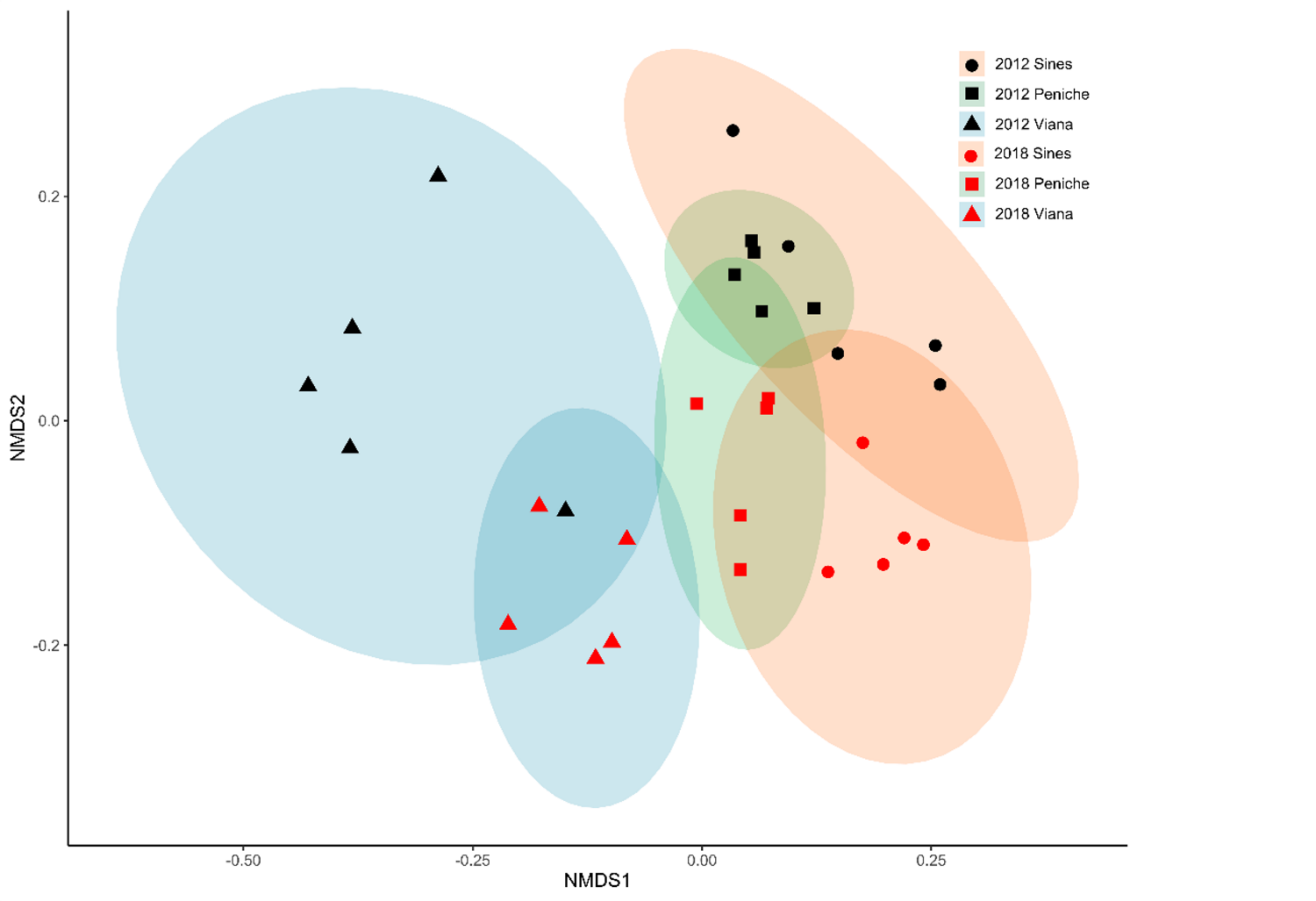
Two-dimensional non-metric multidimensional scaling plot showing similarities in the structure of algal assemblages between reefs within each region over the two sampling years. *NMDS* Non-metric multidimensional scaling.

When considering the complete assemblages’ dataset, i.e. using multivariate analyses of species abundance through Permanova, patterns were similar to those detected using univariate descriptors (Table 2). However, the interaction Time x Region was found to be significant (Pseudo-F_1,24_:33.57, *p*(perm) < 0.001, Table 2). Pairwise comparisons were all significant, preventing the identification of the “true” nature of the interaction. When exploring reefs variability using the multivariate Permadisp procedure for the centroids of each factor (see NDMS in Fig. 3), we found temporal trends in the variability were significant at Peniche where the deviation of the centroids increased in 2018 regarding 2012 (Permadisp F_1,174_: 6.0, *p*(perm): 0.001).Significant values are in bold.

**Table 2:** Permutational multivariate analysis of variance (PERMANOVA) and pairwise comparisons examining the effects of time, region and reef on whole macroalgal assemblages. Significant effects are reported in bold.

Source of variation	df	MS	Pseudo-*F*	*p*	Denominator	
Time (T)	1	5	54.036	**0.001**	Reef (T x R)	
Region (R)	2	6.98	75.498	**0.001**	T x R	
T x R	2	3.1	33.578	**0.001**	Reef (T x R)	
Reef (T x R)	24	0.76	8.281	**0.001**	Residual	
Residual	150	0.09	0.24157			
Pairwise comparisons T x		2012			2018	
R	F.Model	R^2^	p.value	F.Model	R^2^	p.value
Viana x Peniche	22.96	0.283	**0.001**	14.79	0.203	**0.001**
Viana x Sines	14.32	0.198	**0.001**	14.00	0.194	**0.001**
Peniche x Sines	8.99	0.134	**0.01**	9.67	0.142	**0.001**

### Community temperature index (CTI)

**Figure 4:**
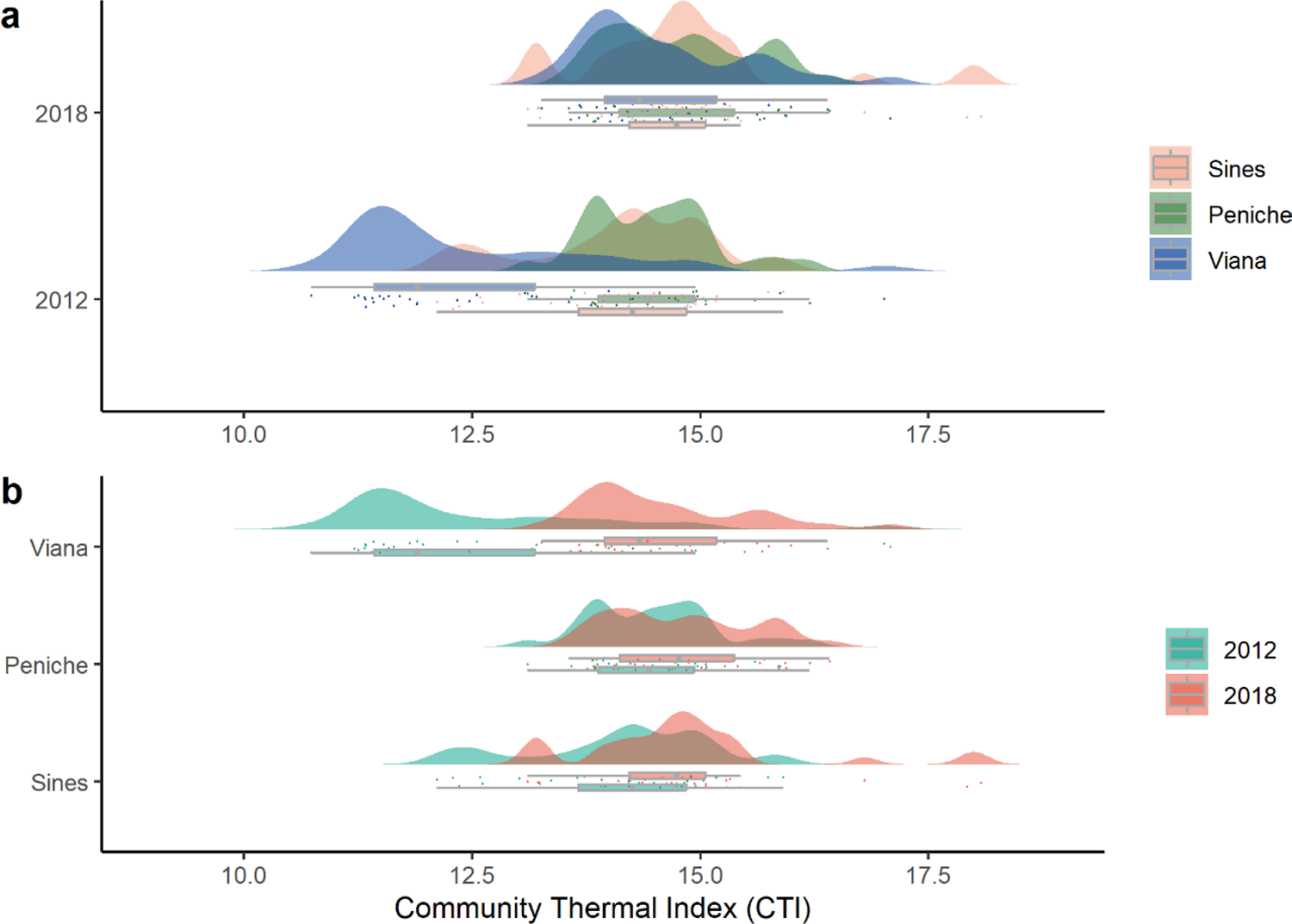
Visual demonstration of the significant effects of interaction between time and region in the community temperature index (CTI): (**a**) differences among regions in each year and (**b**) differences between years in each region on values of CTI (community temperature index). Points correspond to each reef.

**Figure 5:**
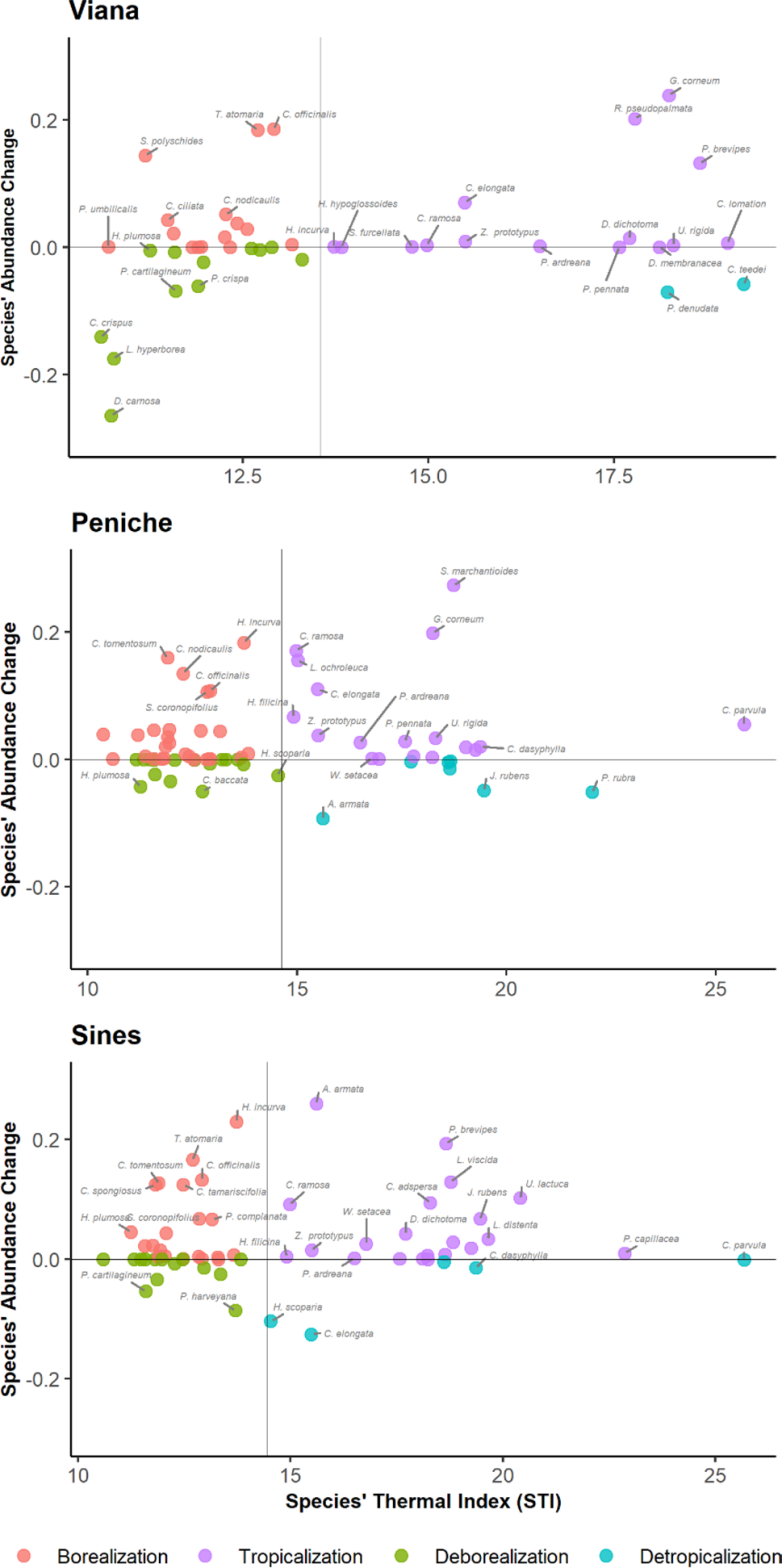
Processes behind the processes of community temperature index changes in each region. Thin horizontal line indicates no change in species abundance and thin vertical line is the mean CTI for each region.

Overall, the community temperature index (CTI) significantly rose from 13.70 ± 0.15 °C (mean ± SE) in 2012 to 14.73 ± 0.10 °C in 2018 (Table 3). Algae communities of Sines (14.12 ± 0.18) and Peniche (14.48 °C ± 0.13) hold significantly higher CTIs than Viana’s assemblages (12.49 ± 0.26 °C) in 2012 (Table 3, Fig. 4a). In 2018, there were no significant differences among communities (Fig. 4a). At the regional level, CTIs increased significantly from 2012 to 2018, in Sines and Viana (Table 3, Fig. 4b). The abundance of warm-affinity species (with STIs higher than mean CTIs) increased in all regions, suggesting that tropicalization is the main driver of changes in the CTIs of each region (Fig. 5). The abundance of cold-affinity species increases in Sines and Peniche, contributing to a moderate increase of the CTIs comparedg with communities in Viana. The processes behind the significant increase of CTIs in Viana were tropicalization and deborealization (decrease in abundance of cold-affinity species) (Fig. 5).

**Table 3:** ANOVA examining the effects of time, region and reef on the community temperature indexes (CTIs). Computed least-squares means for significant factors and factor combinations. * p < 0.05, ** p < 0.01, *** p < 0.001. Significant values in bold. Time x Region a posterior contrasts only include those combinations with significant differences.

Source of variation	df	MS	F value			
Time (T)	1	48.29	90.39 ***			
Region (R)	2	20.53	38.43***			
Time:Region	2	13.58	25.42***			
Reef(T*R)	30	0.53	0.46			
Residual	144	1.18				
Cochran C’ test	C = 0.12	*p*-value = 0.06				
Contrast						
		t-ratio	p-value		t-ratio	p-value
2012–2018		-6.4	< 0.0001	PEN12 - VIA12	7.09	< 0.0001
				PEN18 - VIA12	8.21	< 0.0001
PEN–SIN		0.85	0.67	SIN12 - VIA12	5.83	< 0.0001
PEN–VIA		5.49	< 0.0001	SIN18 - VIA12	8.262	< 0.0001
SIN–VIA		4.63	< 0.0001	VIA12 - VIA18	-7.54	< 0.0001

### Morpho-functional groups abundance

**Figure 6:**
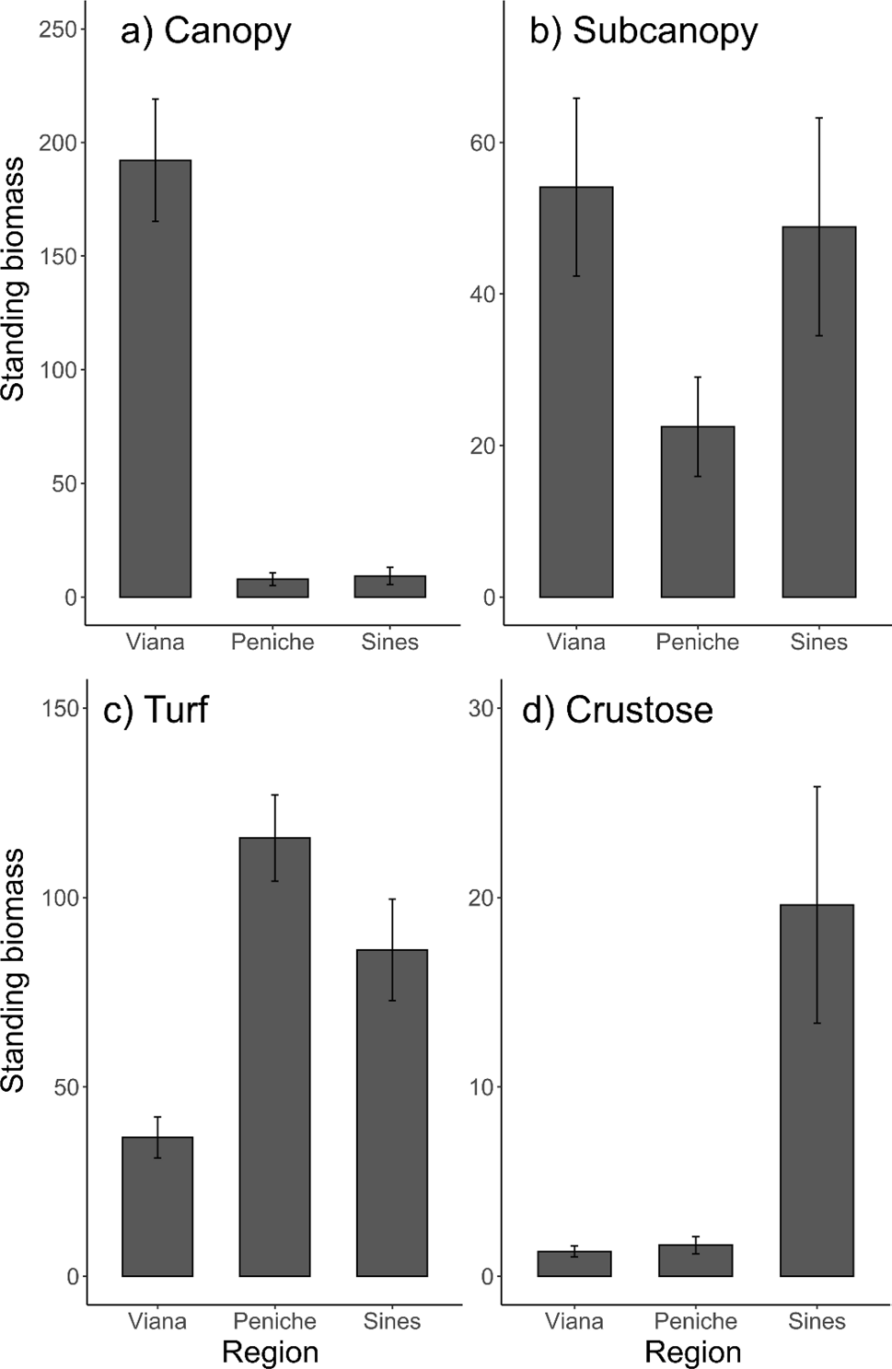
Bar plots to illustrate the significant effects of region on the standing biomass (wet weight g 0.25 m^2^) for the four morpho-functional groups for (**a**) Canopy, (**b**) Subcanopy, (**c**) Turf and (**d**) Crustose. Bars are mean values for the two surveys (2012–2018) ± SE, n = 6.

Regional differences were found for most of the morpho-functional groups (Fig. 6, Table 4), except for sub-canopy species which did not show any latitudinal effect (Fig. 6b, Table 4). Turf species were more abundant in the centre and south regions (Fig. 6c, Table 4) while encrusting species were more abundant in the south (Fig. 6d, Table 4). The north region (Viana) holds a larger amount of canopy algae than the other regions (SNK *p* < 0.001, Fig. 6a, Table 4). Regarding the temporal evolution of the functional groups, only turf species showed a significant change, with an overall increase in biomass from 2012 to 2018 (SNK *p* < 0.01, Table 4).

**Table 4:** ANOVA examining the effects of time, region, and reef on the mean of encrusting species, turf species, subcanopy species and canopy species.

Source of variation	df	Encrusting		Turf		Subcanopy		Canopy	
		MS	*F*	MS	*F*	MS	*F*	MS	*F*
Time (T)	1	124	0.108	412.68	11.6932**	57,221	2.676	2089	0.038
Region (R)	2	6577.5	5.752**	364.43	10.326***	17,225	0.805	674,246	12.451***
(T) x (R)	2	139.8	0.122	28.52	0.8081	10,074	0.471	3837	0.07
Reef (T x R)	24	1143.4	1.547	35.29	3.5149***	21,383	4.121***	54,149	6.075***
Residual	150	738.8		10.04		5188		8912	
Cochran C' test		C = 0.4159, P < 0.001		C = 0.11291, P = 0.107		C = 0.34024, P < 0.001		C = 0.23942, P < 0.001	
Transformation		None		Sqrt(x)		None		None	
SNK test		Region		Time	Region			Region	
		VIA = PEN < SIN		2012 < 2018	VIA < PEN = SIN			PEN = SIN < VIA	

* p < 0.05, ** p < 0.01, *** p < 0.001. Significant values are in bold.

## Discussion

Our surveys conducted between 2012 and 2018 provided valuable insights into the changes occurring in the subtidal seaweed communities along the Portuguese continental coast. The approach goes beyond simply assessing the magnitude of structural changes that occurred. It also aims to shed light on the underlying processes that drive these changes. Our results provide evidence that the macroalgae assemblages are undergoing a fast tropicalization process, which poses a threat to the persistence of Europe's southernmost kelp forests.

One of the most notable findings from our subtidal surveys was the rapid rate at which these assemblages underwent changes within a relatively short time period. Most of the analysed descriptors revealed significant differences between 2012 and 2018, indicating a high rate of the response to climate change in coastal communities^62^. Both, structural and functional changes were detected in the assemblages. The overall species richness and diversity, as indicated by descriptors like the Shannon H’ index, increased over the 6-year period. This increase in species number can be attributed, in part, to the progressive colonization of the Portuguese coast by warm-water affinity species, including non-native species, which inflated local species diversity^63^. Ocean temperature is the key driver controlling geographic distribution of subtidal seaweed species. It is predicted that warmer conditions will result in shifts in species range. A comprehensive modelling projection by García Molinos et al.^64^ suggested that increased regional diversities due to the prevalence of range shift expansions outweighing range contractions, are a common phenomenon under future environmental scenarios. Local–regional signals of increasing species richness in marine systems have been reported as a general trend in a meta-analyses study^65^. Indeed, we found evidence of range expansions, such as the red seaweed *Liagora distenta*, a warm-affinity species Mediterranean specie that was not found in 2012. Also, the arrival and proliferation of non-native species, such as *Symphyocladia marchantioides*. This species from subtropical origin may be favoured by ocean warming because of its higher thermal lethal limits^66^. Since its first record near Lisbon in 2007 (Herbarium MACOI, specimen no 3564, University of Coimbra, PT), *Symphyocladia marchantioides*, has spread and become abundant in Portugal. Future ecological impacts of this introduction are unknown but because of its creeping growth habit, it may locally dominate the substrate probably preventing the settlement and growth of other seaweeds (authors pers. obs.).

The structure of subtidal seaweed communities along the Portuguese coast follows the latitudinal gradient of temperature, consistent with previous observations^16^. Significant differences among regions were found for most of the community descriptors examined, with the only exception of the biomass of sub-canopy function group. As expected, assemblages in the North were dominated by large kelp species (*Laminaria* spp. and *Saccorhiza polyschides).* which exhibited higher biomass but lower species richness. In contrast, the central and southern communities showed high species richness, with the turfs being the dominant functional group. Indeed, there is a north–south latitudinal gradient, with a large biogeographical disruption between northern communities and those present in the central and southern regions. This pattern follows the existing latitudinal thermal gradient^16,25^. Temperature, known as the most relevant physical driver controlling the geographic boundaries of seaweed species^66–68^, decreases with increasing latitude, following a recognized large-scale pattern in physical oceanography. The impact of seawater temperature gradient on the composition of these communities is also evident in the community temperature index (CTI) results.

However, the latitudinal thermal gradient may not be the only cause driving the large differences among regions. The Iberian upwelling system creates unique conditions for the persistence of cold-water species, reinforcing the disruption between north and central-south communities. Reefs from the northern region are subject to the highest intensity of the Iberian upwelling in Portugal^69^. Under upwelling favourable winds (i.e. northerly winds) surface coastal waters are transported offshore and replaced by subsurface cold and nutrient rich seawaters. On the Portuguese coast, the seawater summer temperatures are eased off by persistent upwelling events (from June to September), providing thermal refugia to cold-affinity species like kelps^70,71^. In fact, in recent years, kelp species have disappeared at higher latitudes of the Iberian Peninsula (the Cantabrian Sea^32,35,72^, where the upwelling influence is less intense^70^. There has been some debate regarding the future trends of the Iberian upwelling system, with some early publications predicting increases in its intensity^73,74^ while others suggesting reductions^24,75^. Recent evidence suggests that even under increased upwelling favourable winds, the strengthening of stratification due to ocean warming will counteract the upwelling, resulting in an overall decrease in the upwelling intensity^27^. Whether this scenario is already happening and its consequences for coastal assemblages requires further attention.

The thermal signature of the subtidal seaweed communities measured using the community temperature index (CTI) increased from 2012 to 2018. As expected, seaweed communities of Sines and Peniche were more similar, holding significantly higher CTIs than Viana do Castelo assemblages in 2012. In 2018, there was a significant increase of CTIs in Viana do Castelo, resulting in the absence of differences among regions. This result suggested a trend to biotic homogenization of subtidal communities along the coast of Portugal, a process recognized in many other European seaweeds assemblages^76^. During the period of the study, there was an increase in the abundance of species with warm-water affinity along the coast of Portugal. For instance, the abundance of *Gelidium corneum* in Peniche and Viana do Castelo and *Cryptopleura ramosa* in all regions. This increase in the abundance of warm-affinity species suggests that the observed changes are primarily driven by tropicalization. Similar tropicalization of temperate marine ecosystems has been widely reported in coastal assemblages^9,77^, including along the Portuguese coast for fish communities^78^. In addition to tropicalization, changes in Viana subtidal communities are also associated with a process of deborealization, characterized by the decrease in abundance of cold-affinity species. Despite being less discussed, deboralization often acts synergistically with tropicalization and has profound implications on marine communities^54^. If cold-affinity species are unable to find thermal refuge like that provided by the upwelling, deborealization might become the main process driving changes in subtidal seaweed communities on the northern Portuguese coast.

The last remarkable finding was the rapid functional changes detected in the seaweed assemblages over a six-year period., Turf-forming species significantly increased their biomass in the algal assemblages. This morpho-functional group has been linked to anthropogenic climate change^44,79^. According to Airoldi^44^, turfs are small, fast- growing, opportunistic species with high coverage and turnover rates that can be highly stress tolerant compared to other larger functional groups. Turfs transform deeply marine ecosystems by replacing structurally diverse seaweed canopies with simplified habitats dominated by filamentous algae, which provide limited resources for ecosystem maintenance^32,79–83^. Besides being a bio-indicator of environmental stress, an increase in turf abundance can damage the maintenance of biodiversity of well-structured ecosystems. Turfs provide small three-dimensional structures that increase the rate of sediment build-up in its structure and may inhibit the recruitment of larger algae that require a firm substrate, limiting the recovery of the canopy population^84–86^. This accumulation of sediment was observed particularly in our samples containing the invasive species of turf *Symphyocladia marchantioides*, which grows close to the substrate forming a mat. Under these conditions, it is easy to speculate that turf expansion might imperil the persistence of canopy macroalgae, i.e. marine forest like kelps, even though this study did not show any significant decrease in this functional group.

In conclusion, the coast of north Portugal hosts the southernmost North Atlantic seaweed forests of several cold-adapted species like *Laminaria hyperborea and Saccharina latissima.* Iberian upwelling is probably key in maintaining those northerly communities at these latitudes. However, we confirmed that subtidal macroalgal communities are experiencing major changes at very fast pace. This novel evidence of “fast tropicalization” of the subtidal communities on the Portuguese coast is likely a strong sign of the effects of anthropic climate change over these unique assemblages. The loss of these marine forests would lead to the loss of their key ecological and economic role. A comprehensive understanding of the mechanisms responsible for the observed changes is urgently required for an effective management and protection of these relictual populations, as well for the implementation of successful restoration actions. Simultaneously, monitoring programs like the one presented in this study should provide information about the evolution of these meridional marine assemblages.

## Supplementary Information

Supplementary Information.

## Data Availability

The datasets generated and analysed during the current study is available from figshare, https://doi.org/10.6084/m9.figshare.23585400.
