# Supplementary material for: Rapid tropicalization evidence of subtidal seaweed assemblages along a coastal transitional zone
Source: Sci Rep. 2023 Jul 20;13:11720. doi: 10.1038/s41598-023-38514-x (PMC10359287; doi:10.1038/s41598-023-38514-x)
Supplement: Supplementary file 1 — Supplementary Information. [file 41598_2023_38514_MOESM1_ESM.docx]

**S1. Community temperature index methodology**

**Species data**

Data for each species' occurrence were collected in Ocean Biogeographic Information System (OBIS) (https://obis.org/) and Global Biodiversity Information Facility (GBIF) (https://www.gbif.org/). A total of 424,286 georeferenced observations were retrieved from OBIS and GBIF for 94 species. From these observations, dubious records, possible misidentifications, and probable introductions were removed based on literature, as well as duplicate records within the environmental variables pixel size (5arcmin). To decrease aggregation in observations, records were randomly removed according to the Nearest Neighbour Index (NNI) and using the R package “spatialEco” [1]. The modeling dataset for building the models included 4259 for 94 species.

**Environmental data**

The environmental factors or ecogeographical variables were retrieved from Global Marine Database (Bio-ORACLE [2]) using the R package ‘sdmpredictors’ [3]. A total of 14 EGVs were downloaded for the present conditions (2000 – 2014). EGVs association was tested through Pearson’s correlation test. Accordingly, ten variables (Table S1) were selected for further analyses based on the lowest correlation (r < 0.74). The only exception was the minimum and maximum sea surface temperatures (r = 0.91).

**Table S1:** Environmental variables used to assess the global area of each species’ occupancy.

| **Name** | **Description** | **Units** |
| --- | --- | --- |
| Calcite (mean) | Mean concentration of calcite (CaCO3) | mol/m^3^ |
| Chlorophyll A (minimum) | Minimum concentration of chlorophyll A | mg/m^3^ |
| DA (maximum) | Maximum diffuse attenuation coefficient at 490 nm | m^-1^ |
| pH | Water acidity | unitless |
| PAR (maximum) | Maximum photosynthetically available radiation | E/m^2^/day |
| Phosphate | Reactive ortho-phosphate concentration [HPO_4_^-2^] | µmol/L |
| Nitrate | Dissolved inorganic nitrate and nitrate or nitrite concentration | µmol/L |
| Salinity | Dissolved salt content | PSS |
| SST (maximum) | Maximum sea surface temperature | °C |
| SST (minimum) | Minimum sea surface temperature | °C |

**Ecological niche models**

To assess the global area of each species’ occupancy, ecological Niche Models models (ENMs) were built for each species. Models were built using the Maximum Entropy approach implemented in the R package “Biomod2” [4], and using default parameters, with 5 replicates. They were built with 75 % of the data, and the remaining 25 % was used for testing the models. Individual models were evaluated using the Area under the curve (AUC) of the receiver-operating characteristics (ROC) plot [5] the True Skill Statistic (TSS). TSS has been demonstrated as highly effective for assessing model performance [6], and therefore, only models with TSS higher than 0.70 were kept for further analysis. To assess the global area of occupancy of each species, continuous models were converted into binary maps using the TSS threshold as it maximizes both the number of presences and absences correctly classified by the model [7].

**Community Temperature Index analyses**

To define the thermal “niche” of each species, the maps of the area of occupancy were used to extract the quantiles of the sea surface temperatures, for each species [8]. The median (50th percentile) of temperatures in each species-occupied area was used as the STI [8]. The Community Temperature Index (CTI) was calculated for each locality, rocky reefs, and time (all diving campaigns) as the abundance-weighted mean of all STIs [8, 9], as following:

$$CTI= \frac{\sum_{i=1}^{i=N} {STI}_{i}\omega_{i}}{\sum\omega_{i}}$$

where $\omega_{i}$ is the abundance of each species *i* and N is the number of species in the community.

Finally, the four processes promoting changes in CTI were assessed following the methodology of McLean et al. [10], where (i) tropicalization is an increase in the abundance of warm-affinity species; (ii) borealization is an increase in abundance of cold-affinity species; (iii) de-tropicalization is a decrease in the abundance of warm-affinity species; (iv) and de-borealization is a decrease in the abundance of cold-affinity species). Here, warm-affinity species are those whose STI is higher the mean of community, and the STI of cold-affinity species is lower than the mean [10].

**References:**

1. Evans, J. S., Murphy, M. A. spatialEco. R package version 1.3-6, <https://github.com/jeffreyevans/spatialEco>, (2022).
2. Assis J., Lucas A.V., Bárbara I., Serrão E.Á. (2016). Future climate change is predicted to shift long-term persistence zones in the cold-temperate kelp *Laminaria hyperborea*. *Mar. Environ. Re*. **113**, 174-182 (2021).
3. Bosch, S., Fernandez, S. sdmpredictors: Species Distribution Modelling Predictor Datasets_. R package version 0.2.14, <https://CRAN.R-project.org/package=sdmpredictors> (2022).
4. Thuiller, W., Georges, D., Engler, R., Breiner, F. Package ‘biomod2’: Ensemble platform for species distribution modelling. Functions for species distribution modelling, calibration and evaluation 104pp (2016).
5. Fielding, A. H., & Bell, J. F. A review of methods for the assessment of prediction errors in conservation presence/absence models. *Environ. Conserv*. **24**(1), 38-49 (1997).
6. Allouche, O., Tsoar, A., Kadmon, R. Assessing the accuracy of species distribution models: prevalence, kappa and the true skill statistic (TSS). *J. Appl. Ecol.* **43**,1223-1232 (2006).
7. Vale, C. G., Tarroso, P., Brito, J. C. Predicting species distribution at range margins: testing the effects of study area extent, resolution and threshold selection in Sahara-Sahel transition zone. *Divers. Distrib.* **20**, 20-33 (2014).
8. Burrows, M. T. *et al*. Ocean community warming responses explained by thermal affinities and temperature gradients. *Nat. Clim. Chang.* **9**(12), 959-963 (2019).
9. Burrows, M. T. *et al*. Global-scale species distributions predict temperature-related changes in species composition of rocky shore communities in Britain. *Glob. Chang. Biol.* **26**(4): 2093–2105 (2020).
10. McLean, M. *et al*. Disentangling tropicalization and deborealization in marine ecosystems under climate change. *Curr. Biol.* **31**, 4817-4823.e4815 (2021).

**Table S2**  – List of macroalgal taxa with corresponding biomasses measurement (g wet wt per 0.25 m^2^, mean, SD, n = 30) by year in each region across continental Portugal. Macroalgal taxa are listed by morpho-functional groups. Climate affinity (C.A.: I, warm-water; II, cold-water; III, widely distributed).

| **Taxa** | **Viana do Castelo** | | **Peniche** | | **Sines** | |  |
| --- | --- | --- | --- | --- | --- | --- | --- |
|  | **2012** | **2018** | **2012** | **2018** | **2012** | **2018** | **C.A.** |
|  | **mean±SD** | **mean±SD** | **mean±SD** | **mean±SD** | **mean±SD** | **mean±SD** |  |
| **"Incrustant" group** |  |  |  |  |  |  |  |
| *Agissea harveyana* |  |  | 0.119±0.394 |  | 2.372±4.246 | 0.026±0.142 | III |
| *Codium adhaerens* |  |  |  |  | 19.460±54.563 | 13.497±40.228 | I |
| *Colpomenia peregrina* |  |  |  | 0.043±0.184 |  | 0.097±0.534 | III |
| *Cutleria adspersa* |  |  |  |  | 0.019±0.058 | 2.738±5.297 | I |
| *Peyssonnelia rubra* |  |  | 1.043±3.462 |  |  |  | III |
| *Zanardinia typus* | 1.171±2.464 | 1.454±2.055 | 0.522±1.415 | 1.557±2.745 | 0.348±0.955 | 0.663±1.494 | I |
| **"Turf" group** |  |  |  |  |  |  |  |
| *Acrosorium ciliolatum* |  | 0.096±0.437 | 4.761±3.791 | 3.331±4.129 | 0.062±0.234 | 0.95±2.607 | III |
| *Acrosorium venulosum* |  |  |  |  | 0.003±0.004 |  | III |
| *Aglaothamnion sp.* |  | 0.001±0 | 0.147±0.785 | 0.039±0.206 | 0.003±0.013 | 0.393±1.991 | II |
| *Ahnfeltiopsis devoniensis* | 0.004±0.024 |  |  | 0.006±0.034 |  |  | III |
| *Anotrichium furcellatum* |  |  | 0±0.002 |  |  |  | I |
| *Asparagopsis armata* |  |  | 27.276±40.490 | 6.845±18.562 | 1.079±2.304 | 74.792±97.276 | III |
| *Bonnemaisonia asparagoides* |  |  | 0±0.002 | 0.312±1.676 | 0.926±2.201 | 0.207±0.73 | III |
| *Bornetia secundiflora* |  |  | 0.195±0.994 | 0.08±0.282 | 0.022±0.064 | 0.026±0.144 | I |
| *Callithamnion sp.* |  |  |  |  | 0±0.002 | 0.038±0.147 | III |
| *Carpomitra costata* |  |  | 0.011±0.049 |  |  |  | III |
| *Carradoriella denudata* | 1.686±5.014 | 0.014±0.037 |  | 0.039±0.093 |  | 0.091±0.332 | III |
| **Taxa** | **Viana do Castelo** | | **Peniche** | | **Sines** | |  |
|  | **2012** | **2018** | **2012** | **2018** | **2012** | **2018** | **C.A.** |
|  | **mean±SD** | **mean±SD** | **mean±SD** | **mean±SD** | **mean±SD** | **mean±SD** |  |
| *Ceramium ciliatum* |  |  |  |  | 0.219±0.918 |  | II |
| *Ceramium echionotum* |  |  |  |  | 0.001±0.003 |  | III |
| *Ceramium virgatum* |  |  | 0±0.002 |  |  |  | III |
| *Ceramium sp.* |  | 0.001±0 | 0.001±0.003 | 0.001±0 |  | 0.737±2.322 | III |
| *Champia parvula* |  |  |  | 1.134±3.632 | 0.024±0.093 | 0.008±0.03 | III |
| *Chondracanthus acicularis* |  |  | 0.225±0.608 |  | 0.154±0.186 | 0.284±0.626 | III |
| *Chondria dasyphylla* |  |  |  | 0.323±1.67 | 0.24±0.409 | 0.008±0.031 | III |
| *Chylocladia verticillata* |  |  |  |  |  | 0.067±0.296 | III |
| *Cladophora sp.* |  |  | 0.001±0.003 | 0.191±0.34 | 0.265±0.362 | 0.558±1.38 | III |
| *Cladostephus spongiosus* |  | 0.001±0.004 | 0±0.002 | 0.015±0.082 |  | 4.587±11.319 | III |
| *Compsothamnion thuioides* |  |  |  | 0.001±0.007 |  |  | III |
| *Corallina officinalis* | 0.025±0.139 | 12.431±27.991 |  | 3.446±5.395 | 0.022±0.089 | 5.41±7.359 | III |
| *Cryptonemia palmetta* |  | 0.099±0.47 |  | 0.299±0.967 |  |  | I |
| *Cryptopleura ramosa* | 4.37±8.633 | 4.638±6.791 |  | 9.599±11.847 | 0.033±0.123 | 2.663±7.664 | III |
| *Delesseriaceae* |  |  |  | 0.002±0.01 |  |  | III |
| *Dictyota dichotoma* | 9.726±26.449 | 11.981±15.915 | 2.787±3.664 | 2.586±3.381 | 3.756±4.77 | 7.573±21.959 | III |
| *Drachiella heterocarpa* |  |  |  |  | 0±0.002 |  | II |
| *Drachiella spectabilis* |  |  | 0±0.002 |  |  |  | III |
| *Dilsea carnosa* | 37.722±86.296 |  |  |  |  |  | III |
| *Ellisolandia elongata* |  | 1.645±5.075 | 0.014±0.059 | 3.644±9.369 | 6.004±10.771 | 0.232±1.234 | III |
| *Erythroglossum laciniatum* | 0.11±0.604 |  | 0.017±0.055 |  | 0±0.002 |  | III |
| **Taxa** | **Viana do Castelo** | | **Peniche** | | **Sines** | |  |
|  | **2012** | **2018** | **2012** | **2018** | **2012** | **2018** | **C.A.** |
|  | **mean±SD** | **mean±SD** | **mean±SD** | **mean±SD** | **mean±SD** | **mean±SD** |  |
| *Gastroclonium ovatum* |  |  |  | 0.005±0.027 |  |  | II |
| *Gelidium corneum* | 0.187±0.521 | 31.195±71.916 | 0.006±0.035 | 14.579±55.594 |  | 0.001±0 | III |
| *Gigartina pistillata* | 0.023±0.1 |  |  |  |  |  | I |
| *Griffithsia sp.* |  |  |  |  | 0±0.002 |  | I |
| *Halopithys incurva* |  | 0.014±0.079 | 0.642±2.764 | 19.747±47.606 |  | 22.999±105.214 | I |
| *Halopteris filicina* |  |  |  | 1.522±4.861 | 0±0.002 | 0.06±0.211 | III |
| *Halopteris scoparia* |  |  | 3.136±6.162 | 1.928±5.997 | 7.388±18.292 | 0.989±2.503 | III |
| *Halurus equisetifolius* |  | 0.009±0.05 | 0.03±0.155 | 0.475±1.537 | 0.003±0.009 | 0.071±0.257 | II |
| *Heterosiphonia plumosa* | 0.07±0.383 |  | 0.94±2.085 | 0.068±0.276 | 0.033±0.127 | 0.934±3.55 | III |
| *Hypoglossum hypoglossoides* | 0.022±0.084 | 0.024±0.075 | 0.006±0.026 | 0.133±0.263 | 0±0.002 |  | III |
| *Jania rubens* |  |  | 1.253±3.697 | 0.153±0.702 | 0.146±0.332 | 1.904±4.125 | III |
| *Lomentaria clavellosa* |  |  | 0±0.002 |  |  |  | III |
| *Lychaete pellucida* |  |  | 0.014±0.037 |  | 0.958±2.028 | 2.569±13.881 | I |
| *Myriogramme minuta* |  |  |  | 0.005±0.027 |  |  | III |
| *Monosporus pedicellatus* |  |  | <0.0001±0 |  | 0±0.002 |  | III |
| *Nitophyllum punctatum* |  |  | 0.316±0.945 | 1.513±2.99 | 0.192±0.8 | 0.467±1.962 | III |
| *Osmundea pinnatifida* |  |  |  | 0.006±0.025 | 0.004±0.017 | 0.385±1.808 | III |
| *Phycodrys rubens* |  |  |  | 0.711±2.706 |  |  | II |
| *Plocamium cartilagineum* | 4.444±17.529 | 1.101±2.132 | 45.183±59.1 | 32.127±50.271 | 13.118±22.747 | 5.673±11.934 | III |
| *Polysiphonia sp.* |  |  | 0.049±0.246 | 1.192±4.352 | 1.445±3.596 | 0.027±0.146 | III |
| *Porphyra umbillicallis* |  | 0.01±0.047 |  |  |  |  | III |
| **Taxa** | **Viana do Castelo** | | **Peniche** | | **Sines** | |  |
|  | **2012** | **2018** | **2012** | **2018** | **2012** | **2018** | **C.A.** |
|  | **mean±SD** | **mean±SD** | **mean±SD** | **mean±SD** | **mean±SD** | **mean±SD** |  |
| *Pterocladiella capillacea* |  |  |  |  |  | 0.146±0.802 | II |
| *Pterosiphonia complanata* | 0.046±0.237 | 0.107±0.556 | 5.832±7.997 | 11.555±16.803 | 0.167±0.351 | 1.91±3.24 | III |
| *Pterothamnion plumula* |  |  |  | 0.072±0.356 |  |  | II |
| *Pterothamnion crispum* |  |  |  | <0.0001±0 | <0.0001±0 | 0.059±0.27 | I |
| *Pterothamnion sp.* |  |  |  | <0.0001±0 | <0.0001±0 |  | III |
| *Rhodophyllis sp.* |  |  | 0.792±2.738 |  |  |  | III |
| *Rhodymenia ardissonei* |  |  |  |  |  | 0.041±0.172 | III |
| *Rhodymenia holmesii* | 4.565±10.263 | 3.029±7.515 | 0.614±1.781 |  | 0.002±0.011 |  | III |
| *Schizymenia dubyi* |  | 0.682±3.32 |  | 0.073±0.26 |  |  | II |
| *Scinaia furcellata* | 0.003±0.015 | 0.013±0.073 |  |  |  |  | III |
| *Sphaerococcus coronopifolius* |  |  |  | 3.346±10.123 | 18.894±40.404 | 49.645±115.806 | III |
| *Stenogramma interruptum* |  | 0.243±0.652 |  |  |  |  | III |
| *Symphiocladia marchantioides* |  |  |  | 42.801±57.524 |  |  | III |
| *Symphyocladiella parasitica* |  | 0.001±0.006 | 0±0.002 | 0.115±0.317 |  |  | I |
| *Taonia atomaria* |  | 11.749±30.071 |  | 0.87±2.663 | 0.087±0.380 | 9.846±29.281 | III |
| *Thuretella schousboei* |  |  |  |  |  | 0.477±2.294 | III |
| *Ulva lactuca* |  |  |  |  |  | 3.099±5.161 | III |
| *Ulva rigida* |  | 0.049±0.109 | 0.005±0.020 | 0.588±2.835 |  |  | III |
| *Ulva sp.* |  |  | 0.01±0.048 |  | 0.009±0.037 |  | III |
| *Vertebrata fucoides* |  |  |  | 0.004±0.022 |  |  | III |
| *Xiphosiphonia ardreana* | 0.001±0.007 | 0.021±0.058 | 0.396±0.835 | 1.01±2.597 | 0.002±0.004 | 0.025±0.124 | I |
| **Taxa** | **Viana do Castelo** | | **Peniche** | | **Sines** | |  |
|  | **2012** | **2018** | **2012** | **2018** | **2012** | **2018** | **C.A.** |
|  | **mean±SD** | **mean±SD** | **mean±SD** | **mean±SD** | **mean±SD** | **mean±SD** |  |
| *Xiphosiphonia pennata* |  | 0.004±0.023 | 0±0.002 | 0.481±1.774 |  | 0.005±0.029 | III |
| *Womersleyella setacea* |  |  |  | 0.024±0.133 |  | 0.426±1.139 | III |
| **"Subcanopy" group** |  |  |  |  |  |  |  |
| *Calliblepharis ciliata* |  | 0.799±3.584 | 0.006±0.035 |  |  |  | II |
| *Calliblepharis jubata* |  |  |  |  |  | 0.009±0.048 | II |
| *Chondracanthus teedei* | 2.747±11.492 | 0.680±2.840 |  | 0.227±1.195 | 0.033±0.181 | 0.333±1.223 | III |
| *Chondria coerulescens* | 0.056±0.299 |  |  |  |  |  | III |
| *Chondrus crispus* | 6.175±21.794 | 0.019±0.069 |  | 0.007±0.04 | 0.004±0.020 |  | II |
| *Codium tomentosum* |  | 0.003±0.016 |  | 8.058±18.134 | 0.521±1.688 | 7.795±15.052 | III |
| *Desmarestia ligulata* |  | 0.473±2.593 |  |  |  |  | II |
| *Dictyopteris polypodioides* | 0.448±2.186 | 0.453±1.238 |  |  | 0.009±0.041 | 0.016±0.086 | III |
| *Gynmogongrus crenulatus* | 0.420±2.302 | 0.092±0.282 | 0.006±0.035 |  | 0.003±0.016 | 0.003±0.018 | I |
| *Liagora distenta* |  |  |  |  |  | 0.584±3.001 | I |
| *Liagora viscida* |  |  |  |  | 0.002±0.013 | 4.963±17.301 | I |
| *Phyllophora crispa* | 2.569±6.678 | 0.532±1.400 | 2.099±6.781 | 4.039±9.403 | 0.058±0.186 | 0.106±0.382 | II |
| *Rhodophyllis divaricata* |  |  |  |  | 0.004±0.024 |  | I |
| *Rhodymenia pseudopalmata* | 0.543±2.072 | 24.225±51.215 | 0.072±0.378 | 0.141±0.548 |  |  | III |
| *Spatoglossum solieri* |  |  |  |  | 0.064±0.241 |  | III |
| **"Canopy" group** |  |  |  |  |  |  |  |
| *Gongolaria baccata* |  |  | 1.007±3.055 |  |  |  | II |
| *Gongolaria nodicaulis* |  | 1.042±5.168 |  | 5.459±11.9 | 0.123±0.588 | 0.018±0.099 | I |
| **Taxa** | **Viana do Castelo** | | **Peniche** | | **Sines** | |  |
|  | **2012** | **2018** | **2012** | **2018** | **2012** | **2018** | **C.A.** |
|  | **mean±SD** | **mean±SD** | **mean±SD** | **mean±SD** | **mean±SD** | **mean±SD** |  |
| *Laminaria hyperborea* | 175.850±198.644 | 14.738±58.998 |  |  |  |  | II |
| *Laminaria ochroleuca* |  |  |  | 7.582±29.136 |  |  | II |
| *Phyllariopsis brevipes* |  | 5.209±6.799 | 0.045±0.149 |  | 0.004±0.015 | 13.493±32.891 | I |
| *Saccorhiza polyschides* | 21.612±56.062 | 164.673±187.443 | 0.026±0.142 | 0.743±1.828 |  |  | II |
| *Sargassum muticum* |  | 0.344±1.885 |  | 0.875±4.792 |  | 0.35±1.404 | I |
